# Spontaneous lymphoproliferation differentiates two groups of HTLV-1 asymptomatic carriers: with and without high cell proliferation and death

**DOI:** 10.1007/s12026-026-09826-7

**Published:** 2026-08-19

**Authors:** Marta de Souza Porto, Tatiana Mitiko, Victor Folgosi, Dewton Vasconcelos, Mauricio Domingues, Michel Haziot, Jerusa Smid, Rosa Marcusso, Youko Nukui, Augusto C. P. Oliveira, Tatiane Assone, Jorge Casseb, Gil Benard

**Affiliations:** 1 Laboratory of Medical Investigation in Dermatology and Immunodeficiencies (LIM-56), Tropical Medicine Institute, Department of Dermatology, School of Medicine, University of São Paulo, São Paulo, SP Brazil; 2 Institute of Infectious Diseases “Emílio Ribas”, São Paulo, SP Brazil; 3 Department of Hematology, Hospital das Clínicas, Faculty of Medicine, University of São Paulo (HCFMUSP), São Paulo, SP Brazil

**Keywords:** HTLV-1, Asymptomatic carriers, HAM, Lymphoproliferation, Cell death, Fas and FasL

## Abstract

HTLV-1 infection causes chronic immune activation and a prolonged asymptomatic phase, but the functional features that define early disease remain unclear. We evaluated whether spontaneous proliferation (SP) and related functional immune parameters can distinguish clinical stages across the HTLV-1 spectrum. Spontaneous and mitogen-induced lymphoproliferation (PHA, anti-CD3), measured by CFSE dilution, and in vitro cell death, measured by cytometry, were assessed in 435 HTLV-1-infected individuals: asymptomatic carriers (AC, *n* = 303), individuals with intermediate syndrome (IS, *n* = 21), patients with HTLV-1-associated myelopathy (HAM, *n* = 94), adult T-cell leukemia/lymphoma (ATL, *n* = 17), and two control groups. Notably, SP levels divided the asymptomatic individuals in two subgroups: low-SP (24.5%), similar to the control groups, and high-SP (75.5%), resembling IS, HAM, and ATL patients. Additionally, high-SP AC and HTLV-1 symptomatic groups showed significantly reduced responses to PHA and increased spontaneous and PHA-stimulated cell death, all features also observed in IS, HAM, and ATL patients, while low-SP AC results mirrored those of control groups. This increased cell death is strongly correlated with upregulation of Fas and FasL, especially in lymphocytes from high-SP AC, which may underlie the chronic activation and susceptibility to cell death. These findings suggest that HTLV-1 AC comprises two distinct subgroups: one with high SP and cell death, likely due to ongoing viral activity and immune alterations, and another with low SP, low viral activity, and preserved immune homeostasis. Follow up studies of AC with these functional changes are needed to determine if they can help monitor disease progression and identify asymptomatic individuals at higher risk of clinical deterioration.

**Supplementary Information:**

The online version contains supplementary material available at 10.1007/s12026-026-09826-7.

## Background

HTLV-1 is a deltaretrovirus that primarily infects CD4⁺ T cells, but can also be found in other cell types, including CD8⁺ T lymphocytes, dendritic cells, NK cells, and monocytes [1]. Although most infected individuals remain asymptomatic throughout life, approximately 5–10% develop one of the major associated diseases: Adult T-cell leukemia/lymphoma (ATL) or HTLV-1–associated myelopathy (HAM) [2, 3]. HAM is characterized by chronic inflammation and demyelination of the thoracic spinal cord, resulting in motor impairment and, in some cases, complete loss of mobility in the lower limbs [4]. However, some individuals show slow progression over decades [5–7] or present mild, non-progressive symptoms compatible with an intermediate clinical stage [8].

Although multiple studies have demonstrated correlations between disease progression, inflammatory markers, and proviral load [9–11], diagnosis and clinical monitoring of HTLV-1 infection remain challenging. Current laboratory tools cannot reliably distinguish asymptomatic infection from early disease, and predictive biomarkers of progression are still limited. In this context, functional assays that evaluate alterations in cellular behavior and immune responses provide valuable insights into virus-induced dysregulation.

Spontaneous lymphocyte proliferation is a well-established hallmark of HTLV-1 infection [12–14], reflecting virus-mediated activation and increased turnover of infected clones. This response reflects essential aspects of HTLV-1 biology and HAM pathophysiology, and might provide useful information for monitoring disease-related immune alterations as well as disease outcome. Here, we present data showing that proliferation assays can differentiate the clinical stages of HTLV-1 infection, including two distinct groups of asymptomatic individuals based on their proliferative profile. These groups also showed differential in vitro lymphocyte death rates which correlated with expression of the apoptosis-associated Fas and FasL receptors, suggesting that even asymptomatic carriers may exhibit varying degrees of ongoing subclinical inflammation.

## Methods

### Ethical statements

This study was approved by the Ethics Committees of the Hospital das Clínicas da Faculdade de Medicina da Universidade de São Paulo (CAAE: 74621323.2.0000.0068) and the Instituto de Infectologia Emílio Ribas (CAAE: 86379218.6.1001.0061). All samples were anonymized, and clinical data were obtained from medical records. Clinical trial number: not applicable.

### Patients and controls

The study included fresh, heparinized whole blood samples from the Instituto de Infectologia Emílio Ribas (IIER) and the HC/FMUSP cohorts in São Paulo. Both services are localized in the same health complex and share similar HTLV-1 management guidelines. All the subjects were tested for HTLV-1 using the screening and confirmatory tests recommended by the Ministry of Health of Brazil. The initial test was performed with the ELISA Gold HTLV-1/2 (REM), followed by confirmation with Western Blot (HTLV Blot 2.4 MP Biomedicals, Singapore). Positive samples further underwent a multiplex quantitative real-time PCR (mqPCR) analysis using primers for the Tax and Pol regions [15, 16].

The clinical classification of the HTLV-1-infected patients was determined by a neurologist and a hematologist. Patients were classified into four categories based on the medical history, clinical examination and laboratory data as previously described [8]: Asymptomatic carriers (AC), intermediate syndrome (IS), HTLV-associated myelopathy (HAM), and adult T-cell leukemia/lymphoma (ATL). Individuals with intermediate syndrome (IS) are symptomatic patients who do not fulfill all of the Castro-Costa criteria for HAM. They are classified into this category following a comprehensive clinical evaluation based on a standardized protocol developed by the neurology team at IIER, as described by Marcusso et al. [17]. The management of the two cohorts includes an evaluation of the lymphocyte proliferative capacity. This assessment is performed approximately once a year for symptomatic individuals or every other year for asymptomatic individuals, following a previously described protocol for long-term cohort follow-up [18]. For this study we included all patients who have been on regular follow-up for ≥ 20 years: a total of 435 individuals with confirmed HTLV-1 infection participated. The patient’s most recent immunological assessment was considered in this study. Two comparison groups were studied in parallel: fresh heparinized blood samples were obtained from (a) healthy adults recruited from the institutions’ staff (healthy control group, *n* = 49) and (b) HTLV-1 negative adults with non-T cell immunodeficiencies followed at our service who underwent the lymphoproliferation assay as a screening diagnostic test (NT-ID group, *n* = 53). The NT-ID group comprised patients with predominant humoral immunodeficiency (*n* = 22), innate immunity deficiency [10], myeloid lineage defect [3], autoinflammatory disease [3], complement system [1], others [6], without defined ID at the conclusion of the investigation [8]. Age and gender of the groups are depicted in Table 1.

**Table 1:** Demographic characteristics of study groups

	Control (*n* = 49)	Non-T ID (53)	ASY (303)	IS (21)	HAM (94)	ATLL (17)	*p*
Number female/male^a^	39/10	37/16	215/88	20/1	66/28	10/7	0.11
Age, mean years ± SD^b^	35.9 ± 14.1*	36.6 ± 17.8*	55.8 ± 13.8	57.2 ± 9,7	56.5 ± 13.3	58.5 ± 10.3	< 0.0001*

^a^*p*-value determined through Chi-square test

^b^*p*-value determined through one-way ANOVA with Welch’s correction

^*^significantly different from the HTLV-1 clinical groups

### T-cell proliferation assay

The protocol was adapted from previous publications [19, 20]. Briefly, PBMCs were isolated from heparinized blood samples using Ficoll-Paque Plus™. Immediately thereafter, cells were labeled with 0.75 µM CFSE (CellTrace™, Invitrogen) diluted in pre-warmed serum-free RPMI 1640 medium and incubated at 37 °C for 15 min under gentle agitation, protected from light. The staining reaction was quenched by the addition of cold fetal bovine serum (FBS; Gibco), followed by washing steps with cold RPMI 1640 medium. After labeling, 2 × 10⁵ cells per wel**l** were cultured in round-bottom 96-well plates containing 200µL of RPMI 1640 medium supplemented with 10% human AB serum (Sigma-Aldrich). Cells were incubated under three experimental conditions: medium alone (unstimulated), phytohemagglutinin (PHA; 20 µg/mL; Sigma-Aldrich), or anti-CD3 (0.25 µg/mL; clone OKT3; eBioscience™), with all conditions performed in triplicate. Cultures were maintained at 37 °C in a humidified atmosphere containing 5% CO₂ for 90 h. After culturing, the cells were incubated with LIVE/DEAD™ Fixable Far Red Dead Cells (Invitrogen) for 30 min, protected from light. Lymphoproliferation was assessed by CFSE dilution after acquisition on an LSRFortessa™ flow cytometer (BD Biosciences, USA), with at least 50,000 events recorded per sample. Data were analyzed using FlowJo software (v10.0). Cells were sequentially gated to exclude doublets, followed by selection of viable cells (LIVE/DEAD™) and identification of lymphocytes based on FSC (Forward scatter) versus SSC (Side scatter) parameters; proliferation was then quantified within this population as CFSE⁻ cells (see Supplementary Fig. 1A for gating strategy). Proliferative responses were expressed as delta values, calculated as the percentage of proliferating cells under stimulated conditions (PHA or anti-CD3) minus that observed in unstimulated cultures. All assays were performed by a single investigator blinded to participants’ clinical status.

### Cell culture and cellular staining for Fas and Fas-L expression

For the analysis of Fas and FasL expression, PBMCs were evaluated under four conditions: ex vivo (EV), unstimulated (UNS), stimulated with phytohemagglutinin (PHA; 20 µg/mL, Sigma), and stimulated with anti-CD3 (0.25 µg/mL; clone OKT3; eBioscience™). Cells analyzed ex vivo were subjected to extracellular staining immediately after isolation by Ficoll-Paque. The remaining cells were cultured for 48 h in round-bottom 96-well plates containing 200µL of RPMI 1640 medium supplemented with 10% human AB serum (Sigma-Aldrich). For extracellular staining, cells were transferred to 12 × 75 mm polypropylene tubes, washed with PBS, and incubated for 30 min at 4 °C, protected from light, with anti-CD95/Fas (R718, clone DX2, BD Horizon™) for Fas detection, anti-CD178/FasL (RB613, clone NOK-1, BD OptiBuild™) for Fas ligand (FasL) detection, and Fixable Viability Stain 780 (BD Biosciences™) to identify dead cells. This viability dye functions by covalently binding to free amine groups, which are more accessible in cells with compromised membrane integrity. Following incubation, cells were washed with PBS and resuspended in FACSFlow (BD Biosciences) for acquisition on an LSRFortessa™ flow cytometer (BD Biosciences). A minimum of 150,000 events were recorded per sample. Data were analyzed using FlowJo software (v10.0), according to the gating strategy described in Supplementary Fig. 1B.

### Statistical analyses

Statistical analyses were performed to evaluate differences among study groups. Gender distribution was compared using the Chi-square test. Quantitative variables were analyzed by one-way ANOVA with Welch’s correction for parametric data, or by the Kruskal–Wallis test followed by the two-stage linear step-up procedure of Benjamini, Krieger, and Yekutieli for nonparametric data. For intragroup analyses the paired *t*-test or Wilcoxon test were used. Data normality was assessed using the Shapiro–Wilk test. Correlations were evaluated using Pearson’s test for parametric and Spearman’s test for nonparametric data.

The possible role of the spontaneous lymphoproliferation as a tool for HTLV-1 infection staging was assessed through Receiver Operating Characteristic (ROC). ROC curve analysis was performed to evaluate the ability of spontaneous lymphoproliferation (SP) to discriminate HTLV-1–infected individuals (AC, IS, HAM, ATL; *n* = 435) from uninfected controls (healthy controls and NT-ID; *n* = 102). The binary outcome was defined as infection status (HTLV-1 infected vs. uninfected). The optimal cutoff value was determined using Youden’s index (J = sensitivity + specificity − 1), with additional consideration given to maximizing specificity. To assess stage-dependent performance, the cutoff derived from the primary ROC curve was subsequently applied to each HTLV-1 clinical subgroup. In this analysis, specificity remained constant (as defined by the control group), while sensitivity varied according to disease stage.

All statistical analyses were conducted using GraphPad Prism (v. 9.0); *p*-values < 0.05 were considered statistically significant.

## Results

### Setting the optimal cutoff of spontaneous proliferation by ROC curve analysis

ROC curve analysis indicated that the optimal cutoff for spontaneous lymphoproliferation was 4.27% CFSE^Low^ lymphocytes, yielding a specificity of 89.8% (95% CI: 78.2–95.6%) and a sensitivity of 80.0% (95% CI: 76.0–83.5%), with an overall accuracy of 90.3% (95% CI: 87.5–93.2%) (*p* < 0.0001). Increasing specificity beyond this cutoff reduced sensitivity below 80%. When clinical subgroups were analyzed separately, specificity remained at 89.8%, while sensitivity varied according to disease stage: 75.9% among asymptomatic carriers (AC, *n* = 303), 85.7% in individuals with intermediate syndrome (IS, *n* = 21), 92.6% in patients with HAM/TSP (*n* = 94), and 76.5% in patients with ATL (*n* = 17).

### Lymphoproliferation assay

**Fig. 1:**
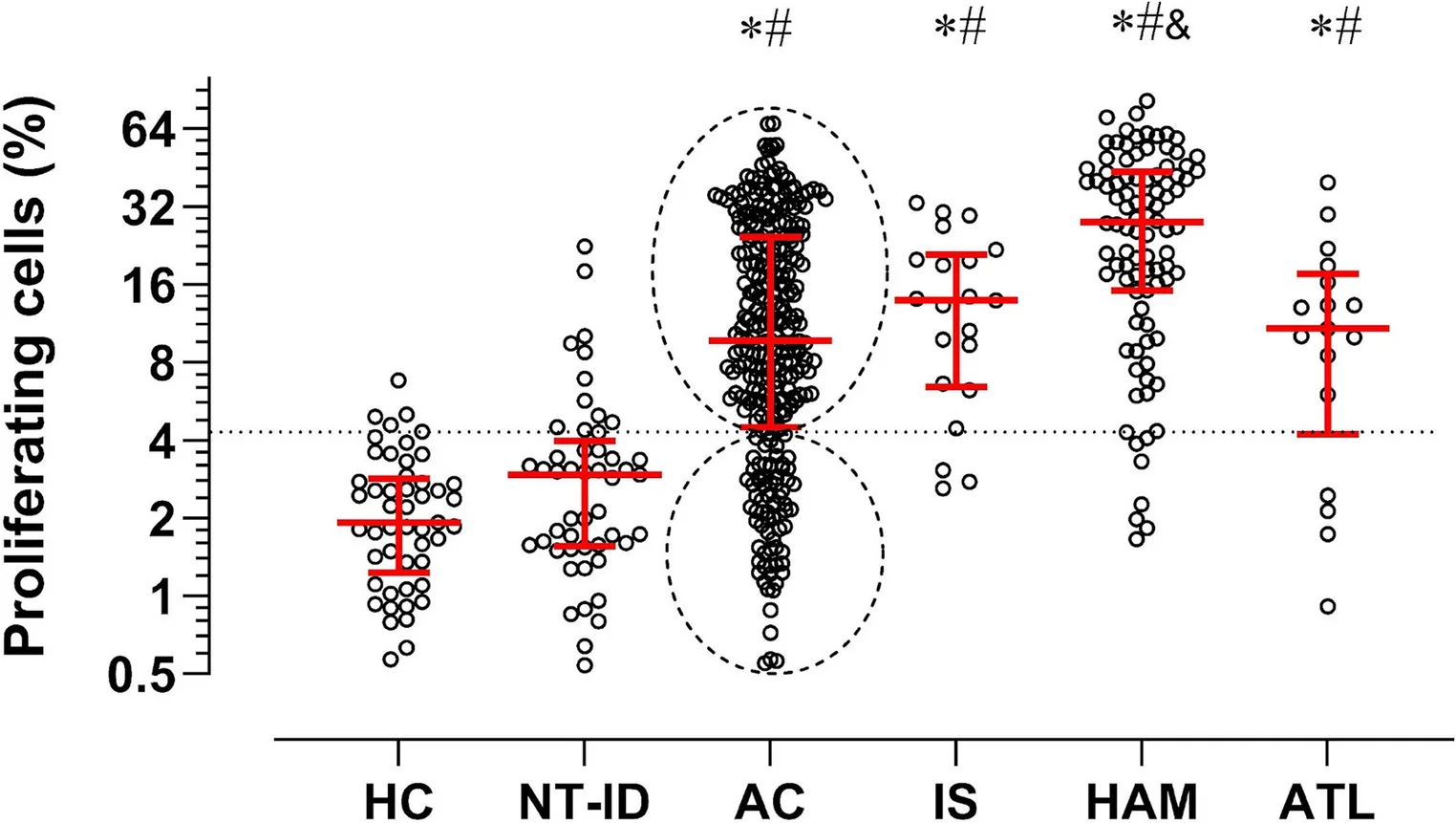
Differences in spontaneous lymphoproliferation among healthy controls (HC), non-T cell immunodeficiency patients (NT-DI) and HTLV-1–infected individuals at different clinical stages (AC, IS, HAM, ATL). Lymphocyte **s**pontaneous proliferation was quantified using CFSE after 90 h of cell culture. Proliferating cells were CFSE(-) Differences were assessed using the Kruskal–Wallis test followed by Benjamini, Krieger and Yekutieli post-test. A Receiver Operating Characteristic (ROC) curve was generated to determine the optimal cutoff discriminating high from low spontaneous proliferation (dotted line). *, significantly different from HC: *p* < 0.0001 for AC, IS, HAM and AT; #, significantly different from NT-ID: *p* < 0.0001 for AC, IS, HAM; *p* < 0.001 for ATL; &, significantly different vs. AC (*p* < 0.0001, IS and ATL (*p* < 0.01). Individual data shown. The circles delimitate the high and low-SP subgroups

Lymphocyte spontaneous proliferation (SP) was assessed in PBMC after 90 h of culture through CFSE staining (Fig. 1). Few subjects in the two control groups presented SP (i.e., more than 4.27% spontaneously proliferating cells): median SP of the healthy and NT-ID control groups were 1,9% (IQR: 1.2–2.8) and 2.9% (IQR: 1.5-4.0), respectively. As expected, all four HTLV infected groups, AC, IS, HAM and ATL showed marked SP. Interestingly, there was a trend for increasing SP from AC to IS and from these to HAM patients. Of note also, as shown in Fig. 1, the application of the ROC-derived cutoff allowed the identification of two AC groups, those with high and those with low SP, corresponding to respectively 75.5% (*n* = 229) and 24.5% (*n* = 74) of the AC group. Levels of SP in the high-SP AC subgroup were not significantly different from those of the IS, HAM and ATL groups, while SP levels of the low-SP subgroup were comparable to the control groups.

**Fig. 2:**
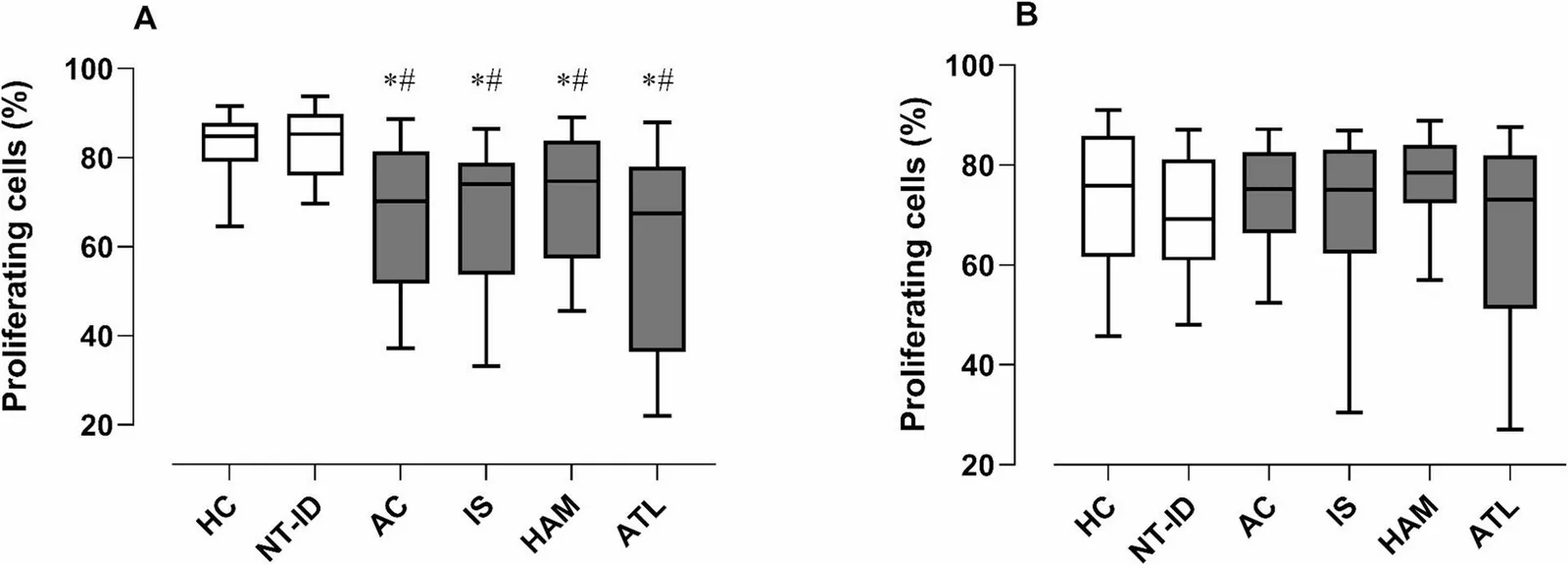
Differential lymphoproliferative response to PHA (**A**) and anti-CD3 (**B**). Lymphocyte proliferation was quantified using CFSE after 90 h of cell culture. Proliferating cells were CFSE(-). Differences were assessed using the Kruskal–Wallis test followed by Benjamini, Krieger and Yekutieli post-test.*: significantly different from HC: *p* < 0.0001 for AC, IS, HAM and ATL; #, significantly different from NT-ID: *p* < 0.0001 for AC, IS, HAM and ATL; There were no statistical differences among the HTLV-1 -infected groups. Medians, IQR and maximum and minimum values shown

We also analyzed the response to the two mitogenic stimuli, PHA and anti-CD3. Lymphocytes from HTLV-1–infected individuals, regardless of the disease stage, exhibited significantly decreased proliferative responses to PHA compared with the control groups, while the responses to anti-CD3 were preserved (Fig. 2).

**Fig. 3:**
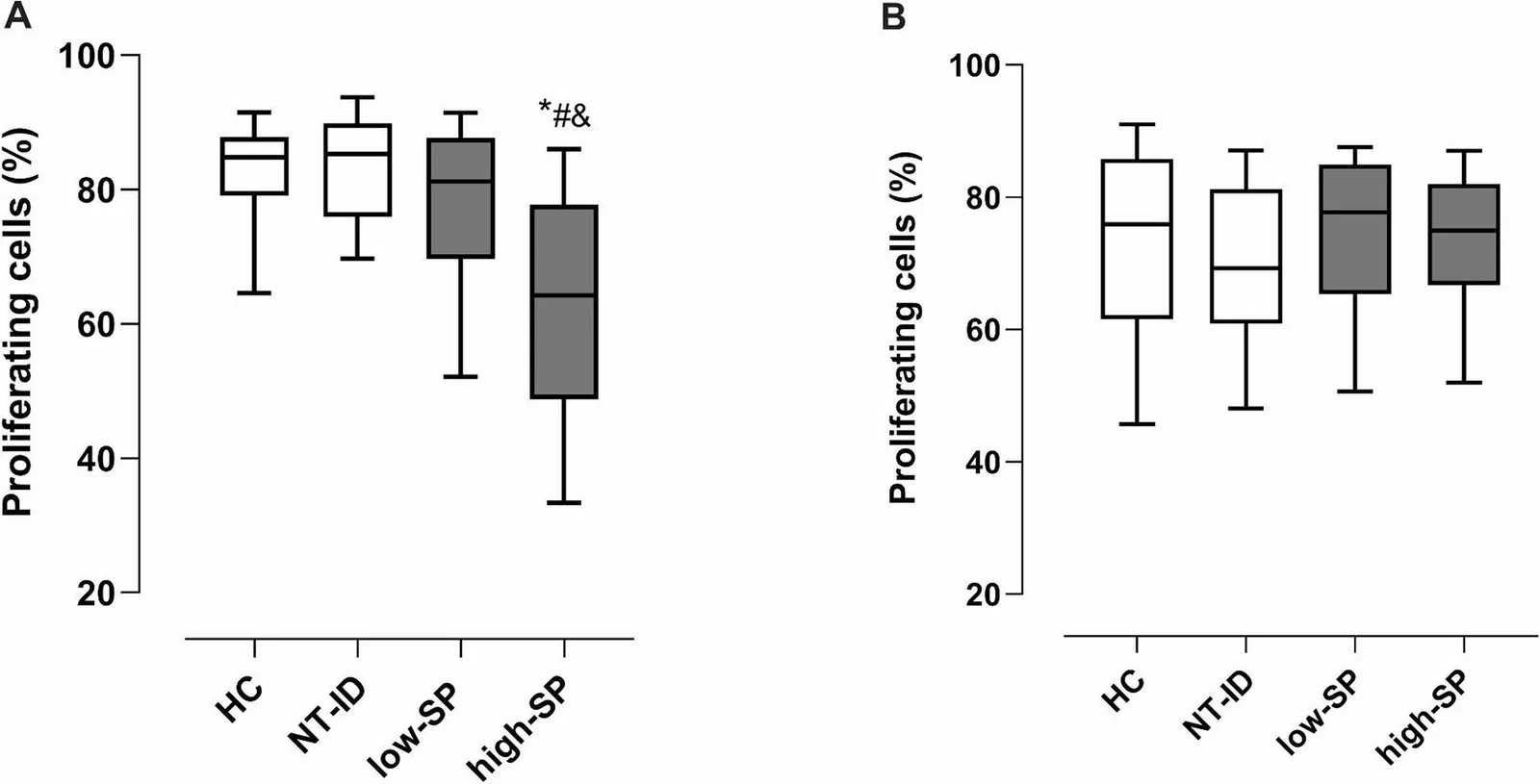
Proliferative responses to PHA (**A**) and anti-CD3 (**B**) in asymptomatic HTLV-1 carriers with low (*n* = 74) and high (*n* = 229) spontaneous proliferation and healthy controls (*n* = 49). Differences were assessed using the Kruskal–Wallis test followed by Benjamini, Krieger and Yekutieli post-test. *, significantly different from HC: *p* < 0.0001); #, significantly different from NT-ID: *p* < 0.0001); &, significantly different from low-SP (*p* < 0.0001). Medians, IQR and maximum and minimum values shown

We then asked whether the AC with low and high SP responded differently to the mitogenic stimuli. The low and high-SP AC groups were comparable regarding age (57.4 ± 13.2 and 60.1 ± 14.8 years-old, respectively) and the predominance of women (72.9% and 69.0%). Figure 3a shows that in fact the high-SP AC had significantly reduced lymphoproliferative responses to PHA compared with the control groups, behaving as the other HTLV-I groups, while the low-SP AC had responses comparable to the control groups. Anti-CD3 responses, on the other hand, did not differ among the groups (Fig. 3b). Thus, SP clearly distinguished two groups of individuals in the AC spectrum of HTLV-I infection.

**Fig. 4:**
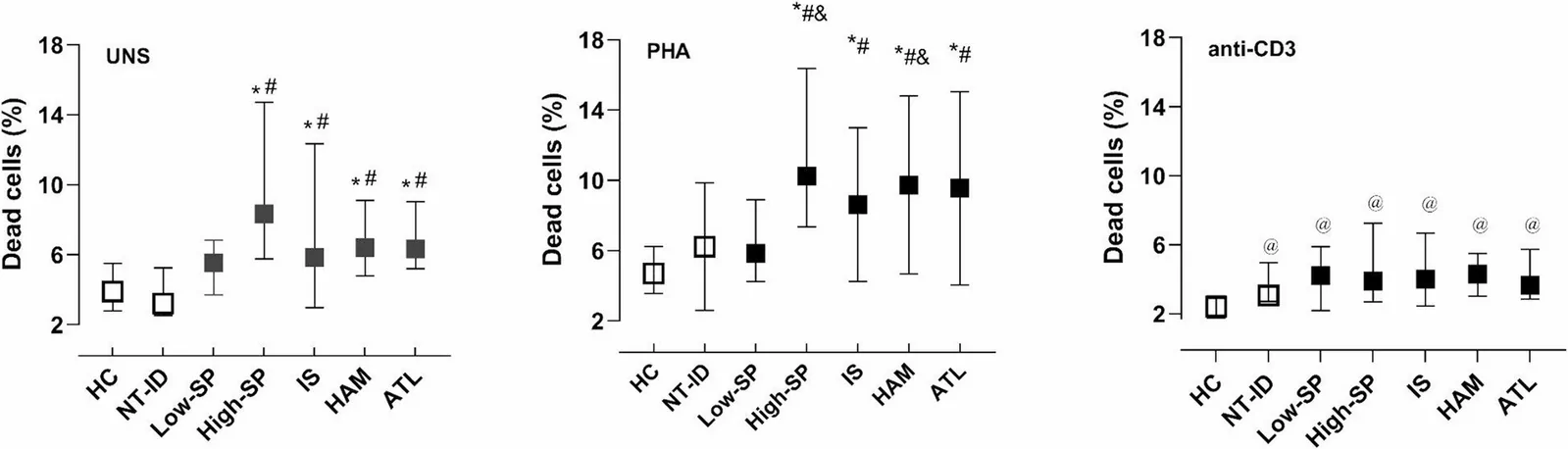
Cell death frequency in lymphocytes after 90 h under stimulated (PHA and anti-CD3) and unstimulated (UNS) conditions in HTLV-1-infected individuals (AC with low or high spontaneous proliferation, IS, HAM, and ATL) and healthy controls (HC). Differences were assessed using the Kruskal–Wallis test followed by Benjamini, Krieger and Yekutieli post-test. *: significantly different from HC: high-SP and HAM, *p* < 0.002; IS and ATL, *p* < 0.05; #: significantly different from NT-ID: high-SP and HAM, *p* < 0.001; IS and ATL, *p* < 0.05; &: significantly different from low-SP: high-SP *p* < 0.001 and HAM *p* < 0.05. @: significantly different from HC: high-SP, low-SP, IS and HAM, *p* < 0.001; ATL and NT-ID, *p* < 0.01. Medians and IQR values shown

To address the previously described hypothesis that virus-associated cellular activation promotes lymphocytes SP [12–14] may also result in increased cell death, we evaluated the frequency of dead cells in the cultures described above using the fluorescence-labeled amine-reactive viability dye succinimidyl ester (LIVE/DEAD™). Although this approach does not allow for the discrimination of the specific mechanisms underlying cell death, it is a useful tool to identify these cells [21]. As shown in Fig. 4, lymphocytes from all HTLV-1 groups, the high-SP AC included, but not the low-SP AC, had significantly increased proportions of dead cells in cultures without exogenous stimulation compared with the two control groups. As a result, the high-SP AC showed significantly increased cell death compared with the low-SP AC. This differential cell death rate was virtually the same in lymphocytes that underwent PHA stimulation: all HTLV groups except the low-SP AC had higher proportions of dead cells compared with the control groups: dead cell number in the latter group was low and similar to that of the control groups. In fact, both the high-SP AC and HAM exhibited significantly higher PHA-induced dead cells frequency than the low-SP AC. Interestingly, in the anti-CD3 stimulated cultures, the levels of dead cells were generally lower than in the other two experimental conditions, with the healthy control group exhibiting the lowest levels among all groups.

To better understand the distinct behavior of the two AC groups, we then analyzed the expression of two cell death associated molecules, Fas and FasL, in a subset of low-SP AC (*n* = 9), high-SP AC (*n* = 10) and healthy donors (*n* = 9).

### Asymptomatic individuals with high spontaneous proliferation have higher expression of pro-apoptotic molecules Fas and FasL

**Fig. 5:**
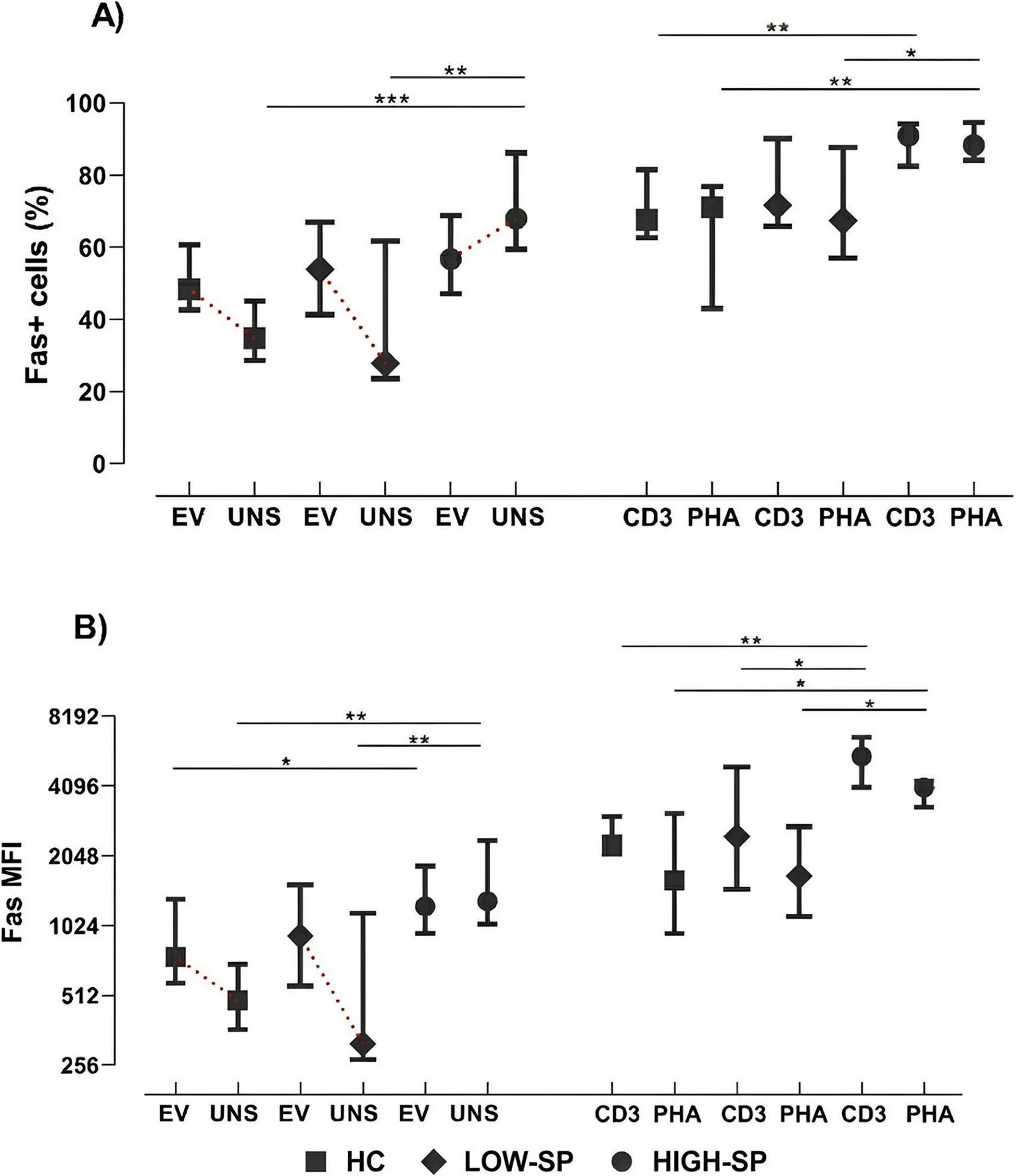
Lymphocytes’ Fas expression in low-SP AC, high-SP AC and HC. ex vivo, unstimulated, anti-CD3 stimulated, and PHA stimulated Fas expression was analyzed based on cell frequency (A) and MFI (B). Differences were assessed using the Kruskal–Wallis test followed by Benjamini, Krieger and Yekutieli post-test. *, *p* < 0.05 ; **, *p* < 0.01. ex vivo vs. unstimulated intra-group results were compared using the paired t-test or Wilcoxon matched pairs test. Dotted red lines denote the statistically significant (*p* < 0.01) decrease in Fas expression with rest in the HC and low-SP groups, whereas in the high-SP there was an increase in the frequency of dead cells. Medians and IQR values shown

Figure 5a shows that, ex vivo (i.e., immediately after the isolation procedure with Ficoll-Paque), Fas was expressed by at least 50% of the lymphocytes in all three groups, with no significant differences between them. However, after the lymphocytes have rested for 48 h in culture, striking differences emerged: while in both the control and low-SP AC groups Fas expression significantly decreased, in the AC-high group its expression increased further, approximately two-fold higher than in the other 2 groups: medians (IQR) of 68.0% (59.5–86.1) vs. 27.8% (23.6–61.7) and 34.8% (28.7–45.2) (*P* < 0.003 for both comparisons). Both PHA and anti-CD3 stimulation resulted in heightened Fas expression: in all groups it was significantly higher than in resting cells, but again the frequency of Fas-expressing cells was significantly higher in the high-SP AC, with > 80% Fas-expressing lymphocytes. Fas MFI (Median fluorescence intensity) analyses mirrored most cell percentage results (Fig. 5b).

**Fig. 6:**
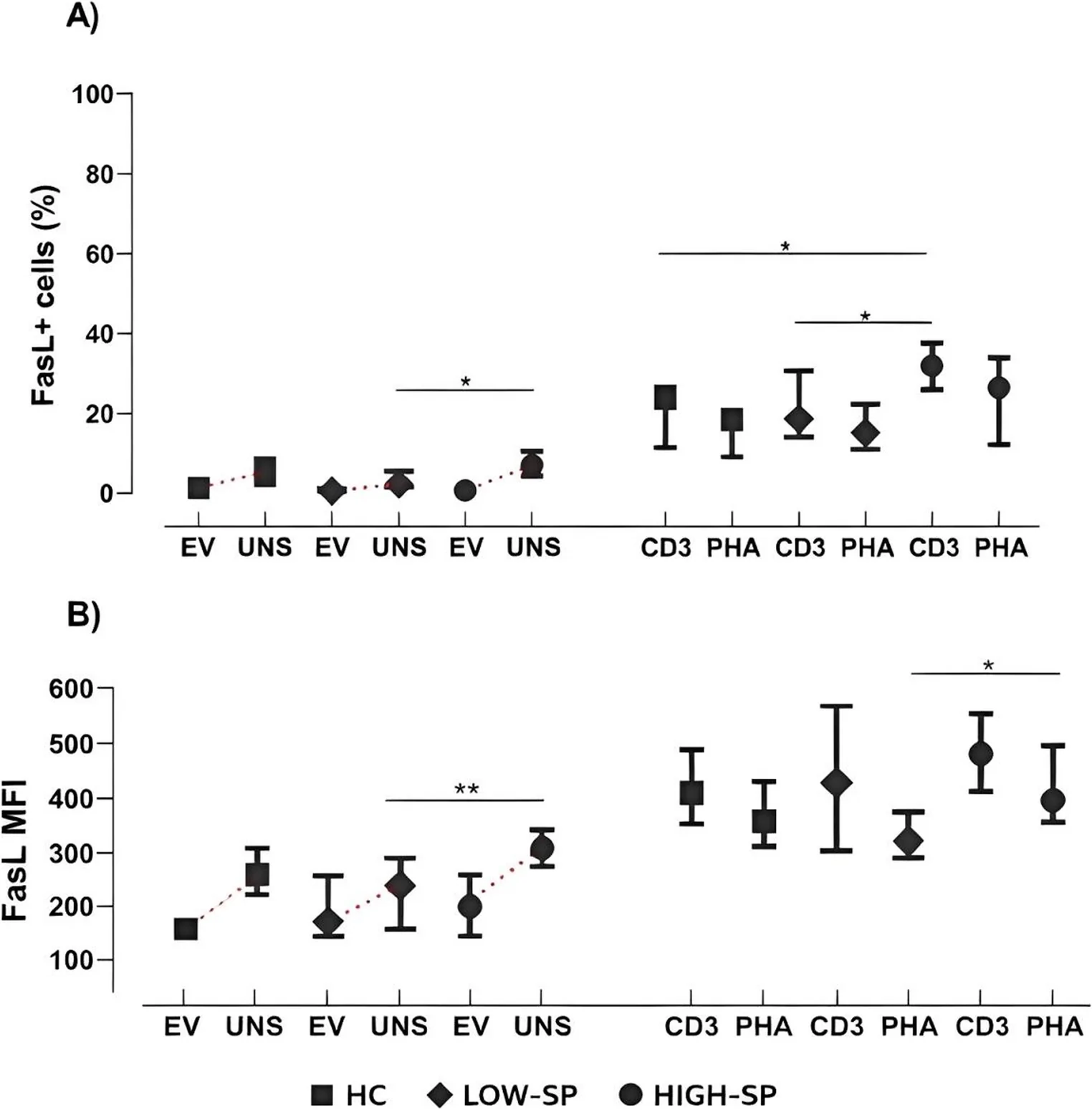
Lymphocytes’ FasL expression in low-SP AC, high-SP AC and HC. ex vivo, unstimulated, anti-CD3 stimulated, and PHA stimulated Fas expression was analyzed based on cell frequency (**A**) and MFI (**B**). Differences were assessed using the Kruskal–Wallis test followed by Benjamini, Krieger and Yekutieli post-test. *, *p* < 0.05 ; **, *p* < 0.01. ex vivo vs. unstimulated intra-group results were compared using the paired t-test or Wilcoxon matched pairs test. Dotted red lines denote the slight but statistically significant (*p* < 0.01) increase in Fas-L expression with rest in the three groups. Medians and IQR values shown

In contrast, the ex vivo frequency of FasL expressing cells was very low across all groups (medians < 1,5%) and only slightly increased in 48 h-resting cells (Fig. 6a). Still, significantly more resting lymphocytes in the high-SP AC expressed FasL compared with the low-SP AC. As expected, FasL expression was more readily detectable in PHA and anti-CD3 stimulated cells in all groups, but the frequency of FasL+ cells in the high-SP group was again higher than in the low-SP AC and control groups. Similar results were observed when the FasL MFI was analyzed (Fig. 6b). Overall, the increased expression of Fas and FasL in the high-SP group, either spontaneously or upon stimulation, suggests that they might play a role in their altered lymphocytes behavior such as their decreased PHA responsiveness and enhanced cell death.

### Correlation between the Fas/FasL expression and cell death and proliferative responses

**Fig. 7:**
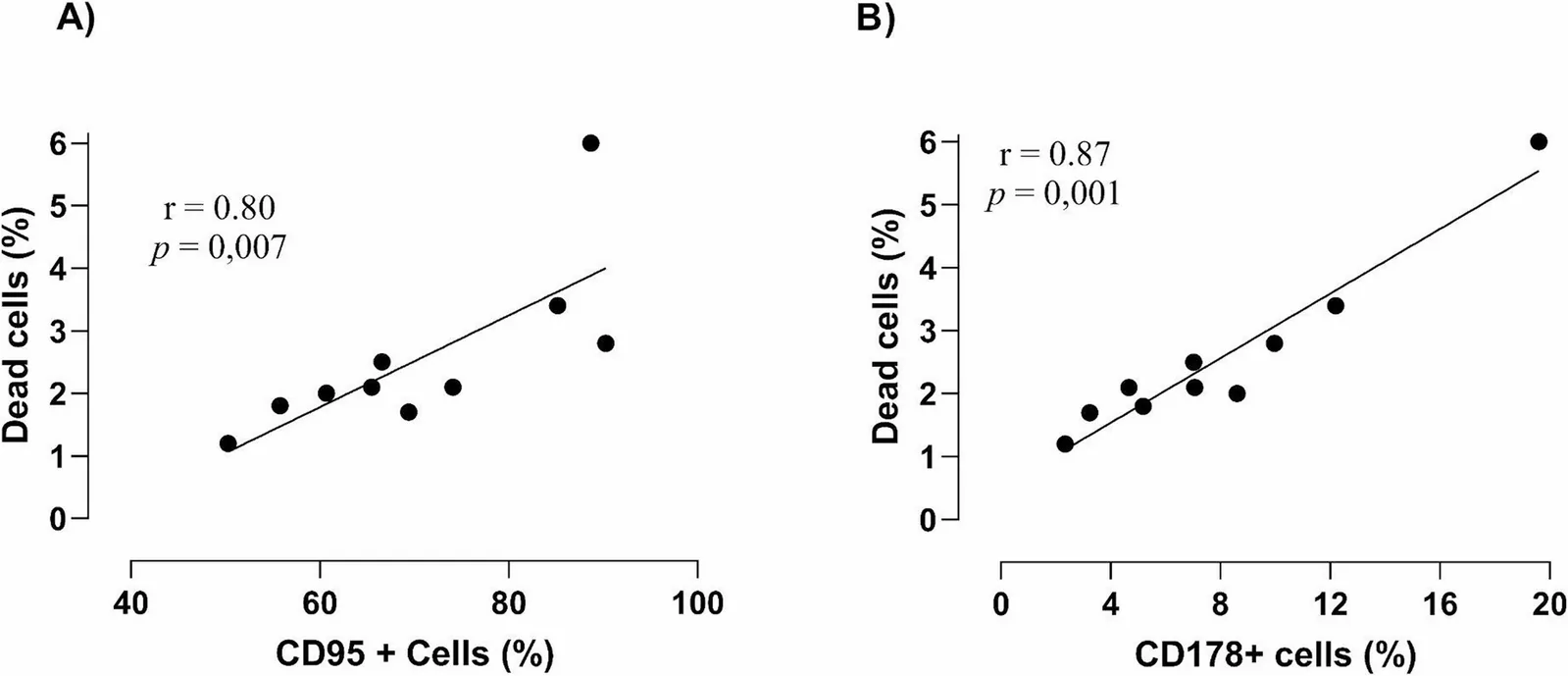
Pearson’s correlation between the frequency of dead cells and Fas/FasL expression in the high-SP AC group. Scatter plots illustrate the relationship between the frequency of dead cells and the expression levels of CD95 (**A**) and CD178 (**B**)

As Fas and FasL expression, depending on the context, may be involved in lymphocyte activation mechanisms rather than apoptosis induction, we then asked whether there was a correlation between Fas or FasL expression and the death rate in the cultured cells (ex vivo data was not analyzed because ex vivo cell death was negligible). With regard to the frequency of Fas+ cells, despite the limited number of patients evaluated, a strong positive correlation was found with the frequency of dead cells in the high-SP CA resting lymphocytes (*r* = 0.80, *p* = 0.007) (Fig. 7a). No such correlation was found in the anti-CD3 or PHA stimulated lymphocytes in this group; this may be due to the narrow high-frequency range of FAS-expressing cells in their stimulated cells: almost all stimulated lymphocytes (anti-CD3 [median (IQR)]: 91.0% (82.4–94.2); PHA: 88.3% (84.1–94.6) expressed Fas (Suppl Fig. 2a and 2b). In the low-SP and control groups no significant correlations were found with Fas+ cells in either unstimulated or stimulated cells, albeit there was a non-significant trend. As the results of the healthy controls and the low-SP AC consistently overlapped, we also performed the analyses pooling the data of the two groups. Significant though weak/moderate positive cell death-Fas correlations were then revealed in all three experimental conditions (0.45 < *r* < 0.6, *p* < 0.05, Suppl Fig. 3a-c).

FasL expression showed an even stronger positive correlation with the frequency of dead cells in resting high-SP AC lymphocytes (*r* = 0.87, *p =* 0.001, Fig. 7b). In PHA-stimulated cells, there was no correlation at all (*p* = 0.65,) while in anti-CD3 stimulated cells, unexpectedly, a negative correlation was found (*r* = -0.73, *p* = 0.016) (Suppl Fig. 2). Differently from Fas, with FasL there were strong correlations in both the low-SP and HC control groups: in resting lymphocytes (*r* = 0.84 and 0.76, respectively) and anti-CD3 stimulated lymphocytes (*r* = 0.72 and 0.77, respectively) (see Suppl Fig. 3 for pooled data). With PHA, correlations showed a trend but did not reach statistical significance (*r* = 0.67 and 0.56, *p* > 0.05), but again the pooled data revealed a moderate correlation (*r* = 0.59, *p* = 0.01).

In contrast, no correlations could be found between Fas or FasL expression and the number of proliferating cells in all groups and experimental conditions, even when the low-SP AC and HC results were pooled (data not shown).

## Discussion

Here we show that ~ 75% of the HTLV-1 infected AC display enhanced lymphocyte SP, and that this functional alteration is accompanied by reduced responsiveness to the mitogen PHA while in vitro lymphocytes’ cell death and Fas/FasL expression were enhanced. We also show that there is a strong association between the percentages of dead cells and Fas/FasL expressing cells in unstimulated lymphocytes of high SP AC.

In fact, the early studies by Itoyama et al. (1989) showing increased SP in HAM patients also found that they had markedly reduced proliferative responses to stimulation with the lectins PHA, Concanavilin, and Pokeweed Mitogen [12]. In this study, they additionally investigated a small group of AC, and showed that they presented mildly but significantly increased SP compared with healthy individuals, but significantly less than that of HAM patients, and no reduced responsiveness to mitogens. Other studies demonstrated reduced lymphoproliferation to recall antigens, more intensely in AC with high SP than low SP, a feature that would underlie the chronic immunosuppression displayed by HTLV-1 infected individuals even in the absence of malignant disease, as evidenced by their increased susceptibility to several infections [22, 23]. However, the impacts of this reduced responsiveness on the HTLV-1 infectious process itself as well on the patients’ disease progression were not addressed. In the present study, taking advantage of the large number of AC studied (*n* = 303), we found that the heightened lymphocyte SP not only differentiated the stages of HTLV-1 infection, as previously suggested by our and other groups [24–26], but that the level of SP also differentiated two subgroups of HTLV-1-infected AC. One AC subgroup had low SP, comparable to our two control groups, one comprising healthy individuals and one comprising patients with suspected immunodeficiencies that do not interfere with T-cell responses (non-T ID). The AC subgroup with high SP, in turn, was comparable to that of the IS, HAM and ATL HTLV-1 clinical groups. Of note was the observation that our NT-ID group presented SP levels similar to the healthy control group, highlighting that the increased SP is a specific feature of the HTLV-1 infection.

We also evaluated in our study groups the lymphoproliferative responses to polyclonal stimulation with PHA and anti-CD3. In agreement with earlier studies [12], IS, HAM e ATL patients proliferated poorly to PHA. Again, the high-SP AC mirrored these patients, presenting reduced responsiveness, while the low-SP AC proliferated as vigorously as the control groups. With anti-CD3, surprisingly, we did not find deficient responses in any of the HTLV-I infected groups. PHA and anti-CD3 stimulation differ in several aspects that might help to explain these differences. PHA is a lectin that binds to N-linked glycans of many lymphocytes membrane glycoproteins, including not only the CD3/TCR complex, but also several other signaling molecules like CD2, CD5, CD45, HLA class I and others, resulting in a clustering of these molecules and the delivery of an activation stimulus through several intracellular signaling pathways [27–29].While PHA induces polyclonal activation of a broader range of cells (including NK and B cells), it is less efficient than anti-CD3 mediated activation, which is able to induce maximal IL-2 secretion by T-cells (30). In fact, it has been shown that PHA-induced early (24–48 h) IL-2 secretion by peripheral blood lymphocytes was markedly lower than that induced with anti-CD3 [30]. In addition, it has been shown that PHA-induced expansion of retrovirally transduced T-cells led to impaired T-cell function, while expansion through CD3 did not [31]. This was explained by the finding that different cell populations were expanded by these stimuli, particularly effector memory T-cells in the case of PHA, which showed an increased apoptosis-prone profile [32]. These observations raised the issue as to whether the SP and the PHA-induced proliferation were associated with enhanced cell death as compared with anti-CD3-induced proliferation.

For this purpose, we assessed the rate of dead cells in the in vitro cultures using a viability marker which marks both apoptotic and nonapoptotic dead cells [21]. In fact, cell death frequency was already higher than controls in unstimulated cells in all HTLV-1 infected groups, except the low-SP AC whose cell death rates were again similar to the control groups. PHA-stimulation resulted in further increase in cell death frequency in all groups, with the same HTLV-1 groups persisting with enhanced cell death rates compared with the controls. Consistent with the previous notion that anti-CD3 stimulation was a more efficient stimulus than PHA [30, 32], cell death frequencies were not only lower than with PHA but also in unstimulated cells. Thus, higher in vitro cell death frequency not only characterized the IS, HAM and ATL patients, but it also distinguished high-SP from low-SP AC. These results suggest the presence of altered baseline activation in HTLV-1-infected T cells, a feature also observed in the high SP subgroup of AC, likely reflecting enhanced viral activity and consistent with the dysregulation of pathways involved in proliferation, survival, and immune activation in these cells [33–35].

To better understand the cell death phenomenon in the low and high-SP AC, we investigated Fas and FasL expression in the in vitro cultured cells of a subset of these patients. These molecules are involved in both T-cell activation and triggering of apoptotic cell death. The results showed that the high-SP AC exhibited a pro-apoptotic phenotype characterized by markedly increased expression of these molecules, in both unstimulated and stimulated lymphocytes, in sharp contrast to the low-SP AC and control groups. Ex vivo, Fas expression was still comparable across groups, consistent with previous reports showing that a substantial fraction of circulating T cells expresses Fas at baseline [36, 37]. Moreover, due to the somewhat toxic effect of Ficoll-Hypaque on cells [38], it is possible that the observed upregulation was due to PBMCs isolation step in all three groups. In fact, after 42 h of rest, Fas levels decreased significantly in control and low-SP AC individuals, whereas they increased further in the high-SP group. The divergence between high and low-SP groups is probably associated with the virus-induced activation in the high-SP AC, in whom Fas upregulation would be sustained even in the absence of exogenous stimuli. Persistent Fas expression has been described as a hallmark of chronically activated T cells in some retroviral contexts [39–41]. Unfortunately, studies on Fas-induced apoptosis in HTLV- 1-infected AC are scarce. Fas/FasL pathway engagement was shown to mediate HTLV-1-tax induced apoptosis in a T cell lineage (Jurkat) [42]. Conversely, in other contexts, HTLV-1 induced pathways were shown to promote in vitro apoptosis resistance [41, 42]. A single study evaluated Fas expression in HTLV-I AC patients, and also found upregulated Fas expression, but this upregulation was not associated with death mechanisms [43]; important methodological differences, however, may underscore the different results, such as the use of fresh cells only in our study.

A pattern similar to Fas emerged for FasL, though its baseline expression was lower than Fas across all groups. FasL expression is highly regulated, being induced only after strong activation signals [43, 44]. The significantly higher levels of FasL in high-SP AC, both in resting and stimulated conditions, indicate that these T cells not only express more Fas but also produce its cognate ligand. The coordinated upregulation of both molecules suggests an increased propensity for AICD, a mechanism critical for T-cell turnover during chronic activation [45, 46]. The correspondence between the frequency and MFI analyses of Fas/FasL underscores the consistency of our findings.

We could not directly measure Fas/FasL-induced apoptotic cell death. However, to address the issue of whether Fas/FasL expression was associated with commitment to death, we performed correlation analyses between dead cells frequency and the number of proliferating cells in high and low-SP AC and in the control group. We found no correlation with proliferating cells in any of the groups and experimental conditions. Conversely, there was strong positive correlation with dead cells frequency in several instances, and this correlation was consistently stronger for FasL than for Fas expression. Notably, in high-SP AC unstimulated cells, the positive correlation with both Fas and FasL was evident but was lost upon anti-CD3 or PHA stimulation, suggesting that virus-induced lymphocyte activation, by itself, enhances Fas and FasL expression [42]. This study has several limitations. We did not address the cell death mechanisms, and the Fas/FasL and correlation studies were performed in a smaller subset of donors; the correlation studies can show associations between phenomena only, but not cause-and-effect relationships. Besides, we measured cell death that may also have happened through non-apoptotic mechanisms, thus the cell death mechanisms could not be addressed. Therefore, mechanistic studies are necessary to characterize the pathways underlying the observed cell death and to clarify the contribution of apoptotic and non-apoptotic mechanisms to our findings. The HTLV-1 groups were older than the control groups; however their mean ages were below 60 years-old and major differences in immunity between young (30–40 y-o) and middle-aged older (less than 60 y-o adults) are not expected [47]; it is worth noting that the differences between the low and high-SP AC groups could not be explained by age differences since they were of the same age (and gender) distribution. The analyses were performed in the total lymphocyte population only: the translation of the observed alterations of spontaneous proliferation, cell death and Fas/FasL expression to specific lymphocyte subsets should be addressed in further studies. However, total lymphocyte analyses provide valuable insights into the natural history of the HTLV-1 infection, given the virus ability to infect multiple cell populations beyond TCD4 lymphocytes, such as B lymphocytes [48]. Finally, we did not use proviral load in this study, because according to our previous studies, as well as studies from other groups, PVL fluctuates along time in the AC subgroup and correlates neither with other inflammatory markers nor with outcome [49–52].

Thus, our study should be regarded as exploratory and further research on low and high-SP AC are warranted, especially those comparing the clinical outcome of the two subgroups of AC. However, differently from previous studies on SP that used a H^3^thymidine-based assay, which only gives a correlate of the number of proliferating cells, our lymphoproliferation assay determined precisely their proportion within the pool of peripheral blood lymphocytes. The finding that many AC presented over 10% of proliferating cells among (unmanipulated) resting lymphocytes signifies that they may be carrying hundreds of millions of circulating activated lymphocytes with a probable inflammatory profile (a healthy adult has up to 4.8 × 10^9^ peripheral blood lymphocytes [53] This is consistent with recent reviews highlighting that, e.g., sustained inflammation is the wildfire contributing to disease progression in HTLV-1 infection [54, 55], as well with a previous study of T cell dynamics showing that T lymphocyte proliferation is elevated in HTLV-1 infected subjects compared with controls [56], resulting in an extra of 10^12^ lymphocytes produced per year. All considered, the data suggest that the virus-induced activation of T cells plays a role in the progression to HTLV-1 associated diseases.

## Supplementary Information

Supplementary Material 1.

## Data Availability

All data supporting the findings of this study are available within the paper and its Supplementary Information. The data that support the findings of this study are available from the corresponding author upon reasonable request.

## References

[1] Richardson JH, Edwards AJ, Cruickshank JK, Rudge P, Dalgleish AG. In vivo cellular tropism of human T-cell leukemia virus type 1. J Virol. 1990;64(11):5682–7. 10.1128/jvi.64.11.5682-5687.1990. 1976827 10.1128/jvi.64.11.5682-5687.1990 PMC248630

[2] Gessain A, Cassar O. Epidemiological aspects and world distribution of HTLV-1 infection. Front Microbiol v. 2012;3:388. 10.3389/fmicb.2012.00388 PMC3498738 23162541

[3] Murphy EL, Hanchard B, Figueroa JP, Gibbs WN, Lofters WS, Campbell M, et al. Modelling the risk of adult T-cell leukemia/lymphoma in persons infected with human T‐lymphotropic virus type I. Int J Cancer. 1989;43(2):250–3. 2917802 10.1002/ijc.2910430214

[4] Osame M, Usuku K, Izumo S, Ijichi N, Amitani H, Igata A, et al. HTLV-I ASSOCIATED MYELOPATHY, A NEW CLINICAL ENTITY. Lancet. 1986;327(8488):1031–2. 10.1016/S0140-6736(86)91298-5. 10.1016/s0140-6736(86)91298-5 2871307

[5] Yamano Y, Sato T. Clinical Pathophysiology of Human T-Lymphotropic Virus-Type 1-Associated Myelopathy/Tropical Spastic Paraparesis. Front Microbiol. 2012;3. 10.3389/fmicb.2012.00389. 10.3389/fmicb.2012.00389 PMC3494083 23162542

[6] Tipismana M, Verdonck K, González E, López G, Clark D, Gotuzzo E. Subacute progression of HTLV-1 associated myelopathy/tropical spastic paraparesis in Peru: a report of 20 cases. BMC Proc. dezembro de. 2008;2(S1):P70, 1753-6561-2-s1-p70.

[7] Nozuma S, Matsuura E, Matsuzaki T, Watanabe O, Kubota R, Izumo S, et al. Familial clusters of HTLV-1-associated myelopathy/tropical spastic paraparesis. PLoS ONE. 2014;9(5):e86144. 24802839 10.1371/journal.pone.0086144 PMC4011969

[8] Haziot ME, Gascon MR, Assone T, Fonseca LAM, Luiz ODC, Smid J, et al. Detection of clinical and neurological signs in apparently asymptomatic HTLV-1 infected carriers: Association with high proviral load. Zunt JR, organizador. PLoS Negl Trop Dis. 2019;13(5):e0006967. 31042700 10.1371/journal.pntd.0006967 PMC6513103

[9] Matsuzaki T, Nakagawa M. HTLV-I proviral load correlates with progression of motor disability in HAM/TSP: Analysis of 239 HAM/TSP patients including 64 patients followed up for 10 years. J Neurovirol. 2001;7(3):228–34. 10.1080/13550280152403272. 11517397 10.1080/13550280152403272

[10] Ariaee N, Abbasnia S, Sabet F, Mirhossein A, Ahmadi Ghezeldasht S, Moshfegh M, et al. HTLV-1 Proviral Load Absolute RT-qPCR Development for Assessing on Clinical Outcomes in HAM/TSP Patients. Rep Biochem Mol Biol. 2023;12(3):393–402. 10.61186/rbmb.12.3.393. 38618262 10.61186/rbmb.12.3.393 PMC11015923

[11] Starling ALB, Martins-Filho OA, Lambertucci JR, Labanca L, De Souza Pereira SR, Teixeira-Carvalho A, et al. Proviral load and the balance of serum cytocines in HTLV-1-asymptomatic infection and in HTLV-1-associated myelopathy/tropical spastic paraparesis (HAM/TSP). Acta Trop. 2013;125(1):75–81. 23022356 10.1016/j.actatropica.2012.09.012

[12] Itoyama Y, Minato S, Kira J, Goto I, Sato H, Okochi K, et al. Spontaneous proliferation of peripheral blood lymphocytes increased in patients with HTLV-I‐associated myelopathy. Neurology. 1988;38(8):1302–1302. 10.1212/WNL.38.8.1302. 2899862 10.1212/wnl.38.8.1302

[13] Pinto LA, Galvão Castro B, Soares MBP, Grassi MFR. An Evaluation of the Spontaneous Proliferation of Peripheral Blood Mononuclear Cells in HTLV-1-Infected Individuals Using Flow Cytometry. ISRN Oncol. 2011;2011:326719. 10.5402/2011/326719. 22191057 10.5402/2011/326719 PMC3236430

[14] Norris PJ, Hirschkorn DF, DeVita DA, Lee TH, Murphy EL. Human T cell leukemia virus type 1 infection drives spontaneous proliferation of natural killer cells. Virulence. 2010;1(1):19–28. 10.4161/viru.1.1.9868. 20640055 10.4161/viru.1.1.9868 PMC2903746

[15] Gonçalves MG, Fukasawa LO, Campos KR, Higa FT, Caterino-de-Araujo A. Development and Validation of Multiplex Quantitative Real-Time PCR Assays for Simultaneous Detection and Differentiation of HTLV-1 and HTLV-2, Using Different PCR Platforms and Reagent Brands. Front Microbiol. 2022;13:831594. 10.3389/fmicb.2022.831594. 35369428 10.3389/fmicb.2022.831594 PMC8965094

[16] Tosswill JHC, Taylor GP, Clewley JP, Weber JN. Quantification of proviral DNA load in human T-cell leukaemia virus type I infections. J Virol Methods. 1998;75(1):21–6. 10.1016/S0166-0934(98)00093-7. 9820571 10.1016/s0166-0934(98)00093-7

[17] Marcusso RM do, Assone N, Haziot T, Smid ME, Folgosi J, Rosadas VA. HTLV-1-Associated Myelopathy (HAM) Incidence in Asymptomatic Carriers and Intermediate Syndrome (IS) Patients. Pathogens. 2024;13(5):403. 10.3390/pathogens13050403. 38787255 10.3390/pathogens13050403 PMC11124065

[18] Castro-Costa CMD, Araújo AQC, Menna-Barreto M, Penalva-de-Oliveira AC. Guia de manejo clínico do paciente com HTLV: aspectos neurológicos. Arq Neuropsiquiatr. 2005;63(2b):548–51. 10.1590/S0004-282X2005000300036. 16059617 10.1590/s0004-282x2005000300036

[19] Quah BJC, Warren HS, Parish CR. Monitoring lymphocyte proliferation in vitro and in vivo with the intracellular fluorescent dye carboxyfluorescein diacetate succinimidyl ester. Nat Protoc. 2007;2(9):2049–56. 10.1038/nprot.2007.296. 17853860 10.1038/nprot.2007.296

[20] Hawkins ED, Hommel M, Turner ML, Battye FL, Markham JF, Hodgkin PD. Measuring lymphocyte proliferation, survival and differentiation using CFSE time-series data. Nat Protoc. 2007;2(9):2057–67. 10.1038/nprot.2007.297. 17853861 10.1038/nprot.2007.297

[21] Perfetto SP, Chattopadhyay PK, Lamoreaux L, Nguyen R, Ambrozak D, Koup RA, et al. Amine reactive dyes: An effective tool to discriminate live and dead cells in polychromatic flow cytometry. J Immunol Methods. 2006;313(1–2):199–208. 10.1016/j.jim.2006.04.007. 16756987 10.1016/j.jim.2006.04.007

[22] Mascarenhas RE, Brodskyn C, Barbosa G, Clarêncio J, Andrade-Filho AS, Figueiroa F, et al. Peripheral Blood Mononuclear Cells from Individuals Infected with Human T-Cell Lymphotropic Virus Type 1 Have a Reduced Capacity To Respond to Recall Antigens. Clin Vaccine Immunol. 2006;13(5):547–52. 10.1128/CVI.13.5.547-552.2006. 16682474 10.1128/CVI.13.5.547-552.2006 PMC1459653

[23] Welles SL, Tachibana N, Okayama A, Shioiri S, Ishihara S, Murai K, et al. Decreased reactivity to PPD among htlv-i carriers in relation to virus and hematologic status. Int J Cancer. 1994;56(3):337–40. 10.1002/ijc.2910560307. 8314320 10.1002/ijc.2910560307

[24] Prates G, Assone T, Corral M, Baldassin MPM, Mitiko T, Silva Sales FC, et al. Prognosis Markers for Monitoring HTLV-1 Neurologic Disease. Neurol Clin Pract. 2021;11(2):134–40. 10.1212/CPJ.0000000000000866. 33842066 10.1212/CPJ.0000000000000866 PMC8032423

[25] Assone T, Kanashiro TM, Baldassin MPM, Paiva A, Haziot ME, Smid J, et al. In vitro basal T-cell proliferation among asymptomatic Human T cell Leukemia Virus type 1 patients co-infected with hepatitis C and/or Human Immunodeficiency Virus type 1. Braz J Infect Dis. 2018;22(2):106–12. 10.1016/j.bjid.2018.02.002. 29499169 10.1016/j.bjid.2018.02.002 PMC9428222

[26] Santos SB, Porto AF, Muniz AL, De Jesus AR, Magalhães E, Melo A, et al. Exacerbated inflammatory cellular immune response characteristics of HAM/TSP is observed in a large proportion of HTLV-I asymptomatic carriers. BMC Infect Dis. 2004;4(1):7. 10.1186/1471-2334-4-7. 15070424 10.1186/1471-2334-4-7 PMC385233

[27] Nagae M, Soga K, Morita-Matsumoto K, Hanashima S, Ikeda A, Yamamoto K, et al. Phytohemagglutinin from Phaseolus vulgaris (PHA-E) displays a novel glycan recognition mode using a common legume lectin fold. Glycobiology. 2014;24(4):368–78. 10.1093/glycob/cwu004. 24436051 10.1093/glycob/cwu004

[28] Lindahlkiessling K, Mattsson A. Mechanism of phytohemagglutinin (PHA) action IV. Effect of some metabolic inhibitors on binding of PHA to lymphocytes and the stimulatory potential of PHA-pretreated cells. Exp Cell Res. 1971;65(2):307–12. 10.1016/0014-4827(71)90006-1. 5554031 10.1016/0014-4827(71)90006-1

[29] Rabinowitz R, Pokroy R, Yu Y, Schlesinger M, Activated Human T-C, Bestow. T-Cell Antigens to Non-T-Cells by Intercellular Antigen Transfer. Hum Immunol. 1998;59(6):331–42. 10.1016/S0198-8859(98)00029-9. 9634195 10.1016/s0198-8859(98)00029-9

[30] Reddy M, Eirikis E, Davis C, Davis HM, Prabhakar U. Comparative analysis of lymphocyte activation marker expression and cytokine secretion profile in stimulated human peripheral blood mononuclear cell cultures: an in vitro model to monitor cellular immune function. J Immunol Methods. 2004;293(1–2):127–42. 10.1016/j.jim.2004.07.006. 15541283 10.1016/j.jim.2004.07.006

[31] Duarte RF, Chen FE, Lowdell MW, Potter MN, Lamana ML, Prentice HG, et al. Functional impairment of human T-lymphocytes following PHA-induced expansion and retroviral transduction: implications for gene therapy. Gene Ther. 2002;9(20):1359–68. 10.1038/sj.gt.3301807. 12365001 10.1038/sj.gt.3301807

[32] Davis L, Vida R, Lipsky PE. Regulation of human T lymphocyte mitogenesis by antibodies to CD3. J Immunoll. 1986;137(12):3758–67. 10.4049/jimmunol.137.12.3758. 3097131

[33] Iwanaga Y, Tsukahara T, Ohashi T, Tanaka Y, Arai M, Nakamura M, et al. Human T-Cell Leukemia Virus Type 1 Tax Protein Abrogates Interleukin-2 Dependence in a Mouse T-Cell Line. J Virol. 1999;73(2):1271–7. 10.1128/JVI.73.2.1271-1277.1999. 9882331 10.1128/jvi.73.2.1271-1277.1999 PMC103950

[34] Hleihel R, Skayneh H, De Thé H, Hermine O, Bazarbachi A. Primary cells from patients with adult T cell leukemia/lymphoma depend on HTLV-1 Tax expression for NF-κB activation and survival. Blood Cancer J. 2023;13(1):67. 10.1038/s41408-023-00841-7. 37137914 10.1038/s41408-023-00841-7 PMC10156663

[35] Zhi H, Yang L, Kuo YL, Ho YK, Shih HM, Giam CZ. NF-κB Hyper-Activation by HTLV-1 Tax Induces Cellular Senescence, but Can Be Alleviated by the Viral Anti-Sense Protein HBZ. Emerman M, organizador. PLoS Pathog. 2011;7(4):e1002025. 10.1371/journal.ppat.1002025. 21552325 10.1371/journal.ppat.1002025 PMC3084201

[36] Mincheff M, Loukinov D, Zoubak S, Hammett M, Meryman H. Fas and Fas Ligand Expression on Human Peripheral Blood Leukocytes. Vox Sang. 1998;74(2):113–21. 10.1046/j.1423-0410.1998.7420113.x. 9501411

[37] Patki AH, Georges DL, Lederman MM. CD4+-T-cell counts, spontaneous apoptosis, and Fas expression in peripheral blood mononuclear cells obtained from human immunodeficiency virus type 1-infected subjects. Clin Diagn Lab Immunol. 1997;4(6):736–41. 10.1128/cdli.4.6.736-741.1997. 9384300 10.1128/cdli.4.6.736-741.1997 PMC170651

[38] Alunni-Fabbroni M, Sandri MT. Circulating tumour cells in clinical practice: Methods of detection and possible characterization. Methods. 2010;50(4):289–97. 10.1016/j.ymeth.2010.01.027. 20116432 10.1016/j.ymeth.2010.01.027

[39] Furukawa Y, Bangham CRM, Taylor GP, Weber JN, Osame M. Frequent reversible membrane damage in peripheral blood B cells in human T cell lymphotropic virus type I (HTLV-I)-associated myelopathy/tropical spastic paraparesis (HAM/TSP). Clin Exp Immunol. 2001;120(2):307–16. 10.1046/j.1365-2249.2000.01211.x. 10.1046/j.1365-2249.2000.01211.x PMC1905651 10792381

[40] Dyrhol-Riise AM, Stent G, Røsok BI, Voltersvik P, Olofsson J, Åsjö B. The Fas/FasL System and T Cell Apoptosis in HIV-1-Infected Lymphoid Tissue during Highly Active Antiretroviral Therapy. Clin Immunol. 2001;101(2):169–79. 10.1006/clim.2001.5101. 11683576 10.1006/clim.2001.5101

[41] Malyshkina A, Littwitz-Salomon E, Sutter K, Zelinskyy G, Windmann S, Schimmer S, et al. Fas Ligand-mediated cytotoxicity of CD4 + T cells during chronic retrovirus infection. Sci Rep. 2017;7(1):7785. 10.1038/s41598-017-08578-7. 28798348 10.1038/s41598-017-08578-7 PMC5552859

[42] Chen X, Liu X, Zdravkovic M, Guo M, Zachar V, Ebbesen P. Role of the Fas/Fas ligand pathway in apoptotic cell death induced by the human T cell lymphotropic virus type I Tax transactivator. J Gen Virol. 1997;78(12):3277–85. 9400978 10.1099/0022-1317-78-12-3277

[43] Zhang J, Gao JX, Salojin K, Shao Q, Grattan M, Meagher C, et al. Regulation of fas ligand expression during activation-induced cell death in T cells by p38 mitogen-activated protein kinase and c-Jun NH2-terminal kinase. J Exp Med. 2000;191(6):1017–30. 10.1084/jem.191.6.1017. 10727463 10.1084/jem.191.6.1017 PMC2193110

[44] Brunner T. Fas (CD95/Apo-1) ligand regulation in T cell homeostasis, cell-mediated cytotoxicity and immune pathology. Semin Immunoll. 2003;15(3):167–76. 10.1016/S1044-5323(03)00035-6. 10.1016/s1044-5323(03)00035-6 14563115

[45] Arkwright PD, Luchetti F, Tour J, Roberts C, Ayub R, Morales AP, et al. Fas stimulation of T lymphocytes promotes rapid intercellular exchange of death signals via membrane nanotubes. Cell Res. 2010;20(1):72–88. 10.1038/cr.2009.112. 19770844 10.1038/cr.2009.112 PMC2822704

[46] Cencioni MT, Santini S, Ruocco G, Borsellino G, De Bardi M, Grasso MG, et al. FAS-ligand regulates differential activation-induced cell death of human T-helper 1 and 17 cells in healthy donors and multiple sclerosis patients. Cell Death Dis. 2015;6(5):e1741–1741. 10.1038/cddis.2015.100. 25950471 10.1038/cddis.2015.100 PMC4669684

[47] Cevirgel A, Shetty SA, Vos M, Nanlohy NM, Beckers L, Bijvank E, et al. Identification of aging-associated immunotypes and immune stability as indicators of post‐vaccination immune activation. Aging Cell. 2022;21(10):e13703. 10.1111/acel.13703. 36081314 10.1111/acel.13703 PMC9577949

[48] Lv A, Fang Y, Lin X, Chen J, Song H, Wang N, et al. B-cell depletion limits HTLV‐1‐infected T‐cell expansion and ameliorate HTLV‐1‐associated myelopathy. Ann Clin Transl Neurol. 2024;11(10):2756–68. 10.1002/acn3.52190. 39186315 10.1002/acn3.52190 PMC11514899

[49] Taylor GP, Tosswill JHC, Matutes E, Daenke S, Hall S, Bain BJ, et al. Prospective Study of HTLV-I Infection in an Initially Asymptomatic Cohort. J Acquir Immune Defic Syndr. 1999;22(1):92. 10.1097/00042560-199909010-00012. 10534152 10.1097/00042560-199909010-00012

[50] Okajima R, Smid J, Duarte AJ, De Oliveira ACP, Casseb J, Sanches JA Junior. Correlation of HTLV-1 proviral load and lymphocyte proliferation from asymptomatic HTLV-1-positive patients and HAM/TSP patients associated or not to skin disorders. Retrovirology. 2014;11(S1):P16–1742.

[51] Demontis MA, Hilburn S, Taylor GP, Human T. Cell Lymphotropic Virus Type 1 Viral Load Variability and Long-Term Trends in Asymptomatic Carriers and in Patients with Human T Cell Lymphotropic Virus Type 1-Related Diseases. AIDS Res Hum Retroviruses. 2013;29(2):359–64. 10.1089/aid.2012.0132. 22894552 10.1089/AID.2012.0132

[52] Ferraz SN, Costa GF, Carneiro Neto JA, Hebert T, De Oliveira CJV, Guerra M, et al. Neurologic, clinical, and immunologic features in a cohort of HTLV-1 carriers with high proviral loads. J Neurovirol. 2020;26(4):520–9. 10.1007/s13365-020-00847-y. 32385802 10.1007/s13365-020-00847-y PMC7438297

[53] Sender R, Weiss Y, Navon Y, Milo I, Azulay N, Keren L, et al. The total mass, number, and distribution of immune cells in the human body. Proc Natl Acad Sci. 2023;120(44):e2308511120. 10.1073/pnas.2308511120. 37871201 10.1073/pnas.2308511120 PMC10623016

[54] Shegefti S, Alaei M, Ghahari N, Telittchenko R, Hanafi SB, Isnard S, et al. Sustained inflammation during human T-lymphotropic virus type 1 infection: a wildfire contributing to disease progression. Front Med. 2025;12:1653384. 10.3389/fmed.2025.1653384. 10.3389/fmed.2025.1653384 PMC12463833 41020241

[55] Miura M, Naito T, Saito M. Current Perspectives in Human T-Cell Leukemia Virus Type 1 Infection and Its Associated Diseases. Front Med. 2022;9:867478. 10.3389/fmed.2022.867478. 10.3389/fmed.2022.867478 PMC9024061 35463007

[56] Asquith B, Zhang Y, Mosley AJ, De Lara CM, Wallace DL, Worth A, et al. *In vivo* T lymphocyte dynamics in humans and the impact of human T-lymphotropic virus 1 infection. Proc Natl Acad Sci. 2007;104(19):8035–40. 10.1073/pnas.0608832104. 17483473 10.1073/pnas.0608832104 PMC1861853

