# Supplementary material for: Spontaneous lymphoproliferation differentiates two groups of HTLV-1 asymptomatic carriers: with and without high cell proliferation and death
Source: Immunol Res. 2026 Aug 19;74(1):90. doi: 10.1007/s12026-026-09826-7 (PMC13490236; doi:10.1007/s12026-026-09826-7)
Supplement: Supplementary file 1 [file 12026_2026_9826_MOESM1_ESM.docx]

**Supplementary figure 1. Gating strategy.** (A) Lymphoproliferation assay. Doublet exclusion was performed by singlet discrimination (FSC-H × FSC-A), followed by identification of the lymphocyte population using SSC-A × FSC-A. Viable cells were selected using Fixable Viability Stain (APC), from which proliferating cells were quantified as the CFSE^low/negative^ population.(B) Apoptosis-marker analysis. Total lymphocytes were first delineated using SSC-A × FSC-H characteristics. Dead cells were excluded based on positivity for Fixable Viability Stain 780, ensuring that Fas/CD95 (R718) and FasL/CD178 (RB613) quantification was restricted to viable events. Marker expression was assessed both as frequency and median fluorescence intensity within this live-cell gate.

**Supplementary Figure 2.**  Correlation between the frequency of dead cells and apoptosis markers in the high-SP AC group. Scatter plots illustrate the relationship between the frequency of dead cells and the expression levels of CD95 (Fas) and CD178 (FasL) in anti-CD3 (A, C) or PHA (B, D) stimulated PBMC cultures. Pearson’s and Spearman’s correlation tests were used for parametric and non-parametric data, respectively.

**Supplementary Figure 3.** Correlation between the frequency of dead cells and apoptosis markers in the low-SP AC and HC groups (pooled data shown: empty dots = HC, black dots = low-SP) . Scatter plots illustrate the relationship between the frequency of dead cells and the expression of CD95 (Fas) and CD178 (FasL) in unstimulated (A and D), anti-CD3 (B and E) and PHA stimulated (C and F) PBMC cultures. Pearson’s and Spearman’s correlation tests were used for parametric and non-parametric data, respectively.
